# Human Keratinocyte Response to Superantigens

**DOI:** 10.1128/mSphere.00803-20

**Published:** 2020-10-07

**Authors:** Patrick M. Schlievert, Francoise A. Gourronc, Donald Y. M. Leung, Aloysius J. Klingelhutz

**Affiliations:** a Department of Microbiology and Immunology, Carver College of Medicine, University of Iowa, Iowa City, Iowa, USA; b Division of Allergy and Immunology, Department of Pediatrics, National Jewish Health, Denver, Colorado, USA; University of Nebraska Medical Center

**Keywords:** RNA-seq, *Staphylococcus aureus*, *Streptococcus pyogenes*, cytokines, keratinocyte, superantigen

## Abstract

Staphylococcus aureus and Streptococcus pyogenes are significant human pathogens, causing infections at multiple body sites, including across the skin. Both are organisms that cause human diseases and secrete superantigens, including toxic shock syndrome toxin-1 (TSST-1), staphylococcal enterotoxins (SEs), and streptococcal pyrogenic exotoxins (SPEs). On the skin, human keratinocytes represent the first cell type to encounter these superantigens. We employed transcriptome sequencing (RNA-seq) to evaluate the human primary keratinocyte response to both TSST-1 and staphylococcal enterotoxin B (SEB) in triplicate analyses. Both superantigens caused large numbers of genes to be up- and downregulated. The genes that exhibited 2-fold differential gene expression compared to vehicle-treated cells, whether up- or downregulated, totaled 5,773 for TSST-1 and 4,320 for SEB. Of these, 4,482 were significantly upregulated by exposure of keratinocytes to TSST-1, whereas 1,291 were downregulated. For SEB, expression levels of 3,785 genes were upregulated, whereas those of 535 were downregulated. There was the expected high overlap in both upregulation (3,412 genes) and downregulation (400 genes). Significantly upregulated genes included those associated with chemokine production, with the possibility of stimulation of inflammation. We also tested an immortalized human keratinocyte line, from a different donor, for chemokine response to four superantigens. TSST-1 and SEB caused production of interleukin-8 (IL-8), MIP-3α, and IL-33. SPEA and SPEC were evaluated for stimulation of expression of IL-8 as a representative chemokine; both stimulated production of IL-8.

**IMPORTANCE** Staphylococcus aureus and Streptococcus pyogenes are common human pathogens, causing infections that include the skin. Both pathogens produce a family of secreted toxins called superantigens, which have been shown to be important in human diseases. The first cell types encountered by superantigens on skin are keratinocytes. Our studies demonstrated, that the human keratinocyte pathway, among other pathways, responds to superantigens with production of chemokines, setting off inflammation. This inflammatory response may be harmful, facilitating opening of the skin barrier.

## Abstract (precis)

Staphylococcus aureus and Streptococcus pyogenes are common human pathogens, causing infections that include the skin. Both pathogens produce a family of secreted toxins called superantigens, which have been shown to be important in human diseases. The first cell types encountered by superantigens on skin are keratinocytes. Our studies demonstrated, that the human keratinocyte pathway, among other pathways, responds to superantigens with production of chemokines, setting off inflammation. This inflammatory response may be harmful, facilitating opening of the skin barrier.

## INTRODUCTION

Staphylococcus aureus and beta-hemolytic streptococci, mainly Streptococcus pyogenes (group A streptococci), commonly cause mucosal and skin infections (1, 2). S. aureus and group A streptococci, all of which cause human infections, produce a large family of pyrogenic toxin superantigens, including toxic shock syndrome toxin-1 (TSST-1), staphylococcal enterotoxins (SEs; for example, staphylococcal enterotoxin B [SEB]), enterotoxin-like molecules, and streptococcal pyrogenic exotoxins (SPEs) (2–7). These superantigens cause the majority of toxic shock syndrome (TSS) cases, among which TSST-1 causes 100% of menstrual TSS (3) cases and TSST-1, SEB, staphylococcal enterotoxin C (SEC), and SPEs, usually SPEA and SPEC, cause nonmenstrual cases (4–9). The association of TSS with these superantigens appears to be a consequence of their high-level production on mucosal and skin surfaces, ranging up to 15 to 20 mg/ml in biofilms (10).

There are numerous other types of skin infections associated with S. aureus besides TSS, including nearly 30 million cases of atopic dermatitis and an additional 30 million cases of diabetes mellitus type II (1, 2, 11). The commonest kinds of S. aureus skin infection can collectively be referred to as “soft tissue” furuncles or abscesses. Both S. aureus and group A streptococci cause impetigo, and group A streptococcal infections in particular can lead to severe invasive diseases, including erysipelas and severe invasive diseases (4, 8).

We have provided a large amount of data related to the penetration of TSST-1 across vaginal mucosal barriers (3, 12, 13). We have coined the phrase “outside-in signaling” to refer to this process (12). TSST-1 interacts with human vaginal epithelial cells (HVECs) solely through the immune costimulatory molecule CD40 to cause “harmful inflammation,” in which chemokines are produced that attract innate and adaptive immune cells into the area of infection, facilitating significant barrier disruption (13). Indeed, the mucosal barriers of both the vaginal and upper respiratory tracts are disrupted in menstrual TSS cases (13, 14). Those prior studies made use of both animal models of vaginal infections and wild-type and isogenic CRISPR-Cas9 knockout HVECs (13).

Despite considerable new information on TSST-1 penetration of mucosal barriers, there is little information on the interactions of S. aureus and group A streptococcal superantigens, represented by TSST-1, SEB, SPEA, and SPEC, with human keratinocytes, and the consequences of such interactions. In this study, we performed transcriptome sequencing (RNA-seq) analysis of TSST-1 and SEB interactions with primary keratinocytes in triplicate.

We also examined the interactions of TSST-1, SEB, SPEA, and SPEC superantigens with isolated, immortalized human keratinocytes from a different donor.

## RESULTS

### RNA-seq analysis of primary human keratinocytes exposed to TSST-1 and SEB.

In order to assess possible effects of superantigens on primary human keratinocytes, the cells were exposed in triplicate to a single dose of TSST-1 and SEB for 6 h. TSST-1 is related to SPEC (2, 7). SEB is related to SEC and SPEA (2, 7). These five superantigens are the major causes of TSS (2–7).

**FIG 1:**
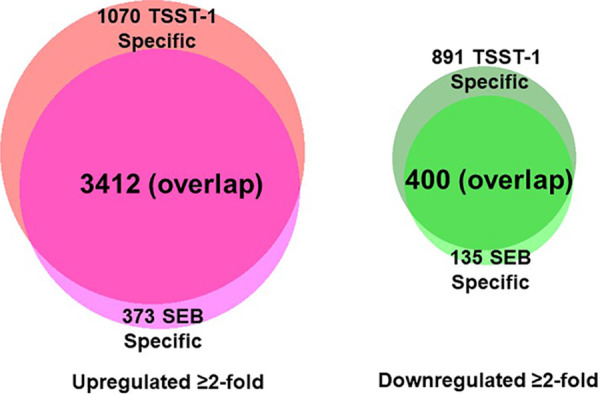
Venn diagram showing total numbers of genes whose expression was up- or downregulated by ≥2-fold in primary human keratinocytes exposed to TSST-1 or SEB as determined by RNA-seq. Shades of red indicate upregulated genes. Shades of green indicate downregulated genes. TSST-1 and SEB were used at 100 μg/ml in triplicate assays with exposure times of 6 h.

RNA-seq analysis was performed with primary keratinocyte exposure to TSST-1 and SEB as representative superantigens. Lists of specific genes whose expression was significantly altered by TSST-1 or SEB are provided at https://www.ncbi.nlm.nih.gov/geo/query/acc.cgi?acc=GSE155775. The overall data obtained are summarized in Fig. 1. The data showed that both superantigens caused surprisingly large numbers of genes to be up- and downregulated. The genes that exhibited 2-fold differential expression (DE) at a significant level (*P* < 0.05) compared to vehicle-treated cells, whether up- or downregulated, totaled 5,773 for TSST-1 and 4,320 for SEB. Of these, 4,482 were significantly upregulated by exposure of keratinocytes to TSST-1, whereas 1,291 were downregulated. For SEB, the expression levels of 3,785 genes were upregulated, whereas 535 were downregulated. There was the expected high overlap in both upregulation (3,412 genes) and downregulation (400 genes).

Table 1 provides a summary of the pathways which were uniquely altered by TSST-1 compared to SEB.

**TABLE 1:** Ingenuity pathway analysis of genes that are altered by TSST-1 compared to SEB

Canonical pathway or molecular and cellular function(s)	*P* value(s)
Role of tissue factor in cancer	2.64 × 10^−3^
Airway pathology in chronic obstructive pulmonary disease	2.68 × 10^−3^
HMGB1 signaling	5.17 × 10^−3^
CXCR4 signaling	5.29 × 10^−3^
Regulation of the epithelial-to-mesenchymal transition by growth factor pathway	6.65 × 10^−3^
Cellular development	9.2 × 10^−3^ to 1.4 × 10^−8^
Cellular growth and function	9.2 × 10^−3^ to 1.4 × 10^−8^
Cell cycle	7.9 × 10^−3^ to 3.2 × 10^−6^
Cell-to-cell signaling and interaction	8.3 × 10^−3^ to 7.6 × 10^−6^

**FIG 2:**
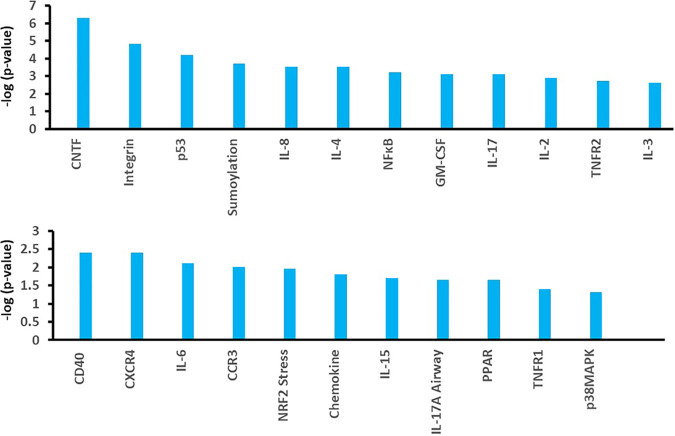
Major pathways significantly altered by exposure of primary human keratinocytes to TSST-1 as determined by RNA-seq. TSST-1 was used at 100 μg/ml in triplicate assays with exposure time of 6 h. PPAR, peroxisome proliferator-activated receptor; GM-CSF, granulocyte-macrophage colony-stimulating factor; p38MAPK, p38 mitogen-activated protein kinase.

**FIG 3:**
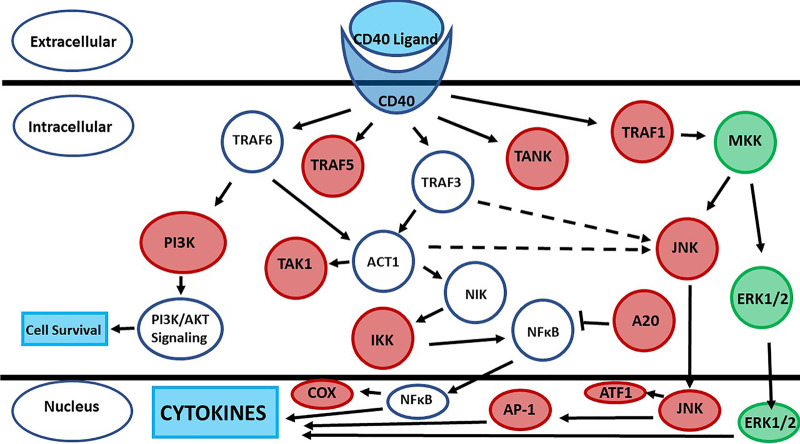
CD40 pathway genes upregulated (red) or downregulated (green) by exposure of primary human keratinocytes to TSST-1 (100 μg/ml) or SEB (100 μg/ml) in triplicate assays for 6 h exposure time. PI3K, phosphatidylinositol 3-kinase; IKK, IκB kinase; JNK, Jun N-terminal protein kinase; ERK1/2, extracellular signal-regulated kinase 1/2.

**FIG 4:**
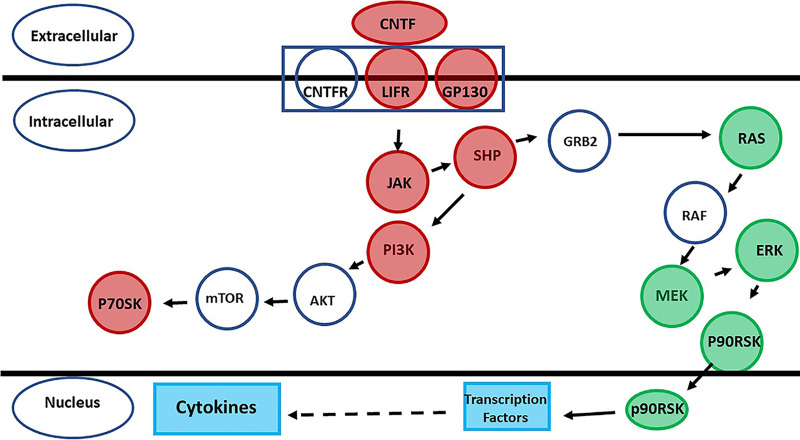
CNTF pathway genes upregulated (red) or downregulated (green) by exposure of primary human keratinocytes to TSST-1 (100 μg/ml) or SEB (100 μg/ml) in triplicate assays for 6 h exposure time.

Our prior studies suggested that TSST-1 and SEB solely use CD40 to initiate chemokine production in HVECs (13). This is the only superantigen receptor on those cells. However, another study has shown that gp130 may be a receptor for SEA on human adipocytes (15), raising the possibility of a second superantigen receptor on some human cell types. On the basis of those prior data, we performed pathway analyses of the primary human keratinocyte response to TSST-1 and SEB. The pathways with statistically significant gene expression are illustrated in Fig. 2, as shown for TSST-1 effects. Of the pathways affected, and as expected, 17 of 23 showed altered cytokine production and/or altered activity of immune cells. Along with other pathways, both the ciliary neurotrophic factor (CNTF) (gp130 pathway) and CD40 pathways were significantly altered. The changes in gene expression that resulted from superantigen effects on the CD40 pathway are shown in Fig. 3 and those for the CNTF pathway in Fig. 4. In these diagrams, red indicates the genes that were significantly upregulated by the superantigens, and green represents the genes that were significantly downregulated. Although the gene for CD40 itself was not significantly altered, many of the downstream genes in the pathway to cytokine production were upregulated. In the CNTF pathway, both CNTF and gp130 genes were significantly upregulated. The CNTF pathway can be activated through interleukin-6 (IL-6). We did not see alteration (up- or downregulation) of IL-6, but we did see upregulation of the IL-6 receptor (IL6R) and signal transducer (IL6ST). Thus, activation of the CNTF pathway must have been occurring by an alternative mechanism.

### Chemokine production by immortalized human keratinocytes exposed to the representative superantigens.

The human skin is one of the most important barriers to S. aureus and group A streptococcal infections. However, both organisms are common causes of skin infections, usually as the result of some underlying condition or prior damage to the skin. Infections of the skin are usually characterized by inflammation. Since we had shown previously that HVECs are active participants in mucosal infections involving superantigens, our hypothesis was that human keratinocytes may function comparably, with chemokine responses leading to harmful inflammation and possible enhancement of infection.

**FIG 5:**
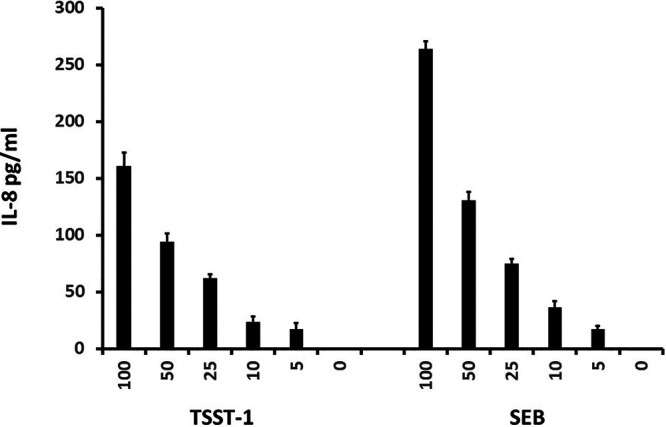
IL-8 production (pg/ml) by immortalized human keratinocytes exposed to TSST-1 or SEB for 6 h in keratinocyte serum-free medium. TSST-1 and SEB doses used ranged from 0 to 100 μg/ml. Values are means ± standard deviations (SD). The *P* value for all doses of both superantigens was <0.001 compared to the no-superantigen wells by Student's *t* test.

From the RNA-seq data obtained with the use of human keratinocytes, many pathways leading to chemokines were altered (Fig. 2). Thus, immortalized human keratinocytes, collected from a donor different from the donor used in the RNA-seq study, showed a dose-dependent response to both TSST-1 and SEB, with production of the chemokine IL-8 (Fig. 5). We used IL-8 because of our prior studies showing that this chemokine has high stability in media (16) compared to other chemokines.

**FIG 6:**
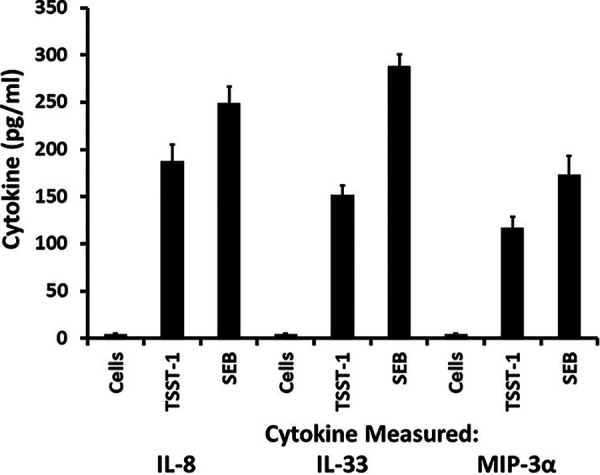
IL-8, IL-33, and MIP-3α production (pg/ml) by immortalized human keratinocytes exposed to TSST-1 or SEB for 6 h in keratinocyte serum-free medium. The TSST-1 and SEB doses used were 100 μg/ml. Values are means ± SD. The *P* value for both superantigens compared to no-superantigen controls, and for all three cytokines, was <0.001 by Student's *t* test.

We then tested production of three chemokines, IL-8, MIP-3α, and IL-33, under conditions of exposure to a single dose of TSST-1 and SEB (Fig. 6). Production of all three chemokines was upregulated by both superantigens; the response seen with SEB was slightly greater that that seen with TSST-1. This is interesting because the response seen with HVECs was the opposite, with the TSST-1 response being stronger than the SEB response; TSST-1 is the only superantigen that causes menstrual, vagina-associated TSS (3, 13). IL-8 is known to attract polymorphonuclear leukocytes (PMNs) to sites of infection (17, 18). MIP-3α represents a sign of damage to the barrier and attracts all cells of the immune system (17, 18). IL-33 is a cytokine thought to be important for skewing responses of T cells in favor of Th2, with downstream IgE production and type I hypersensitivity (19).

**FIG 7:**
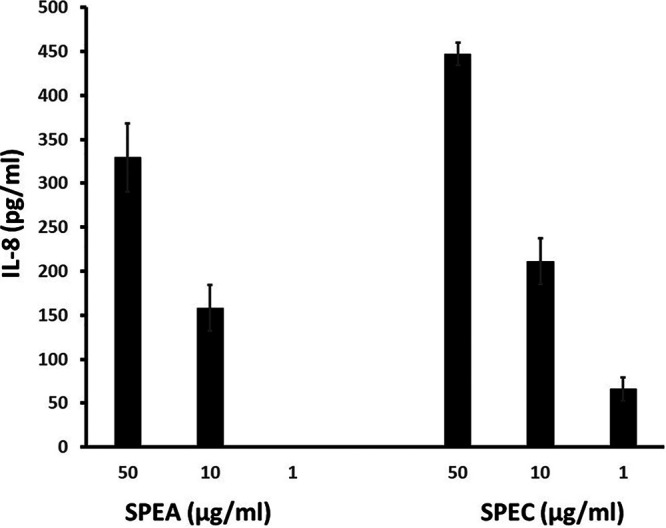
IL-8 production (pg/ml) by immortalized human keratinocytes exposed to SPEA or SPEC for 6 h in keratinocyte serum-free medium. The SPEA and SPEC doses used ranged from 0 to 50 μg/ml. Values are means ± SD. The *P* value for all doses of both superantigens was <0.001 compared to the no-superantigen wells by Student's *t* test.

Finally, we tested the IL-8 response of the same immortalized keratinocytes to the group A streptococcal superantigens SPEA and SPEC (Fig. 7). As with TSST-1 and SEB, the keratinocytes responded in a dose-dependent way. SPEC was somewhat more active than SPEA.

## DISCUSSION

We previously examined the response of HVECs to S. aureus and superantigens (13, 20). With both infections and superantigens, the final targets are cells of the adaptive immune system, importantly, CD4 and CD8 T cells. In the case of S. aureus and its superantigen TSST-1, this leads to massive activation of CD4 T cells and macrophages, sometimes referred to as a “cytokine storm,” but manifesting as menstrual, vaginal TSS.

In order to cause the infections described above, TSST-1 must penetrate the vaginal mucosa. Menstrual, vaginal TSS is characterized by penetration of TSST-1, without significant penetration of the vaginal barrier by S. aureus. The penetration of the vaginal mucosa by TSST-1 has been referred to as “outside-in” signaling (12). This means this agent interacts with epithelial cells to induce harmful inflammation, ultimately opening the barrier to TSST-1 to induce disease (13, 20).

Our studies recently showed that TSST-1 and SEB, as representative superantigens, stimulate HVECs to produce cytokines, primarily chemokines, solely through use of the immune costimulatory molecule CD40 (13). In those studies, TSST-1 was approximately 10 times more potent than SEB in causing chemokine production by HVECs. This may explain the unique association of TSST-1 with menstrual, vaginal TSS. However, another study showed that other cells which are not considered traditional immune cells, such as adipocytes, also bind to SEA at least (15). The associated receptor was identified as gp130, a part of the CNTF pathway. We have not studied this receptor interaction with TSST-1 or other SEs on adipocytes. However, we were able to fulfill Koch’s postulates and the required clinical features of diabetes mellitus type II in rabbits with the use of subcutaneously implanted mini-osmotic pumps containing TSST-1 (11). Additionally, we induced insulin resistance in isolated rabbit adipocytes. These data suggested that adipocytes contain CD40 or gp130 or both CD40 and gp130.

As described in the current manuscript, we initiated studies with RNA-seq to evaluate the human primary keratinocyte response to both TSST-1 and SEB. There were, surprisingly, thousands of dysregulated genes, with most being upregulated and many involving cytokine or immune cell pathways (Fig. 2). These numbers are similar to what we found with HVECs exposed to TSST-1, where we observed 4,858 genes up- or downregulated by TSST-1. However, there were some differences. More genes were upregulated than downregulated for keratinocytes, whereas more genes were downregulated than upregulated for HVECs. Additionally, the sole receptor on HVECs for TSST-1 and SEB was CD40. However, we saw alteration in both the CD40 and CNTF pathways with keratinocytes. We did not see upregulation of CD40 itself, but we did see upregulation of many downstream genes. For the CNTF pathway, we saw upregulation of the genes for both CNTF and gp130, consistent with the prior data for SEA interaction with adipocytes through gp130 (15). It is thus likely that there are both CD40 and CNTF receptors on keratinocytes for superantigens; this does not rule out the involvement of other receptors on keratinocytes based on the large number of genes dysregulated. This potentially large number of other pathways altered by both TSST-1 and SEB will be explored in future studies by us and others. For example, there were pathways whose alteration affected keratinocyte survival, particularly by TSST-1. Such effects suggest that superantigens may impact cancer development. Superantigens are notorious for their massive stimulation of T lymphocytes, but our current and prior (20) studies suggest that HVECs and keratinocytes are also stimulated by superantigens. Previous studies have noted the association of superantigens with severe nasal polyposis (21) and cutaneous T cell lymphoma (22–24). Although both of these conditions may depend on superantigen effects on T lymphocytes, the effects of superantigens on other cell types, as related to cancer development, should be explored.

Not surprisingly, there was a large degree of overlap in the genes altered by TSST-1 or SEB. This might be expected since they have similar structures. For example, both superantigens have only a low-affinity major histocompatibility complex II (MHC-II) binding site in the O/B-fold of the proteins (2, 7). They also share 15 amino acids in the same place in space, which allows accurate folding of the molecules into their defined structures (25).

There are two major differences between TSST-1 and SEB. The T cell receptor (TCR) binding site of TSST-1, as viewed in the standard position, is along the top back of the superantigens, whereas for SEB the T cell receptor binding site is along the top front of the superantigen (7, 25). Additionally, SEB has an emetic cystine loop that is not present in TSST-1 (7, 25); this loop of amino acids is thought to be important for emetic activity of SEB (26). TSST-1 is not emetic (27). It will be of interest in future studies to determine if any of the genes that are uniquely altered by one superantigen or the other are related to specific structural differences in the proteins.

Like the vaginal mucosa, an important function of the skin and thus of keratinocytes is to keep potential pathogens out. However, it is well known that patients with skin conditions such as atopic dermatitis and patients with diabetes mellitus have chronic S. aureus infections (2). All pathogenic S. aureus strains produce one or more superantigens on the skin (as well as on mucosal surfaces) (2, 7, 28, 29), thus suggesting that these secreted proteins are important for human skin diseases. The first major cell type encountered by pathogenic S. aureus and group A streptococcal strains on the skin is the keratinocyte. As with mucosa, it is possible that superantigen disruption of keratinocyte function may be critical in causing disease on that surface. The current studies suggest that there are at least two receptors for superantigens on keratinocytes leading to upregulation of cytokine pathways and immune cell function and thus possibly to harmful inflammation. This suggests that the” outside-in” mechanism of disease causation may function at skin surfaces as well as at mucosal barriers.

The typical effect of a superantigen on cells of the adaptive immune system is massive CD4 T cell proliferation, with skewing of the T cell numbers toward those cells with T cell receptors bearing the variable parts of the β-chains (TCR-Vβs) capable of binding the superantigen. For example, TSST-1 binds only to TCR-Vβ2, whereas SPEC binds to both TCR-Vβ2 and TCR-Vβ8 (30). In a patient with menstrual TSS, TCR-Vβ2 cells may comprise 70% to 80% of the patient’s T cells during acute disease (31). This massive expansion of CD4 T cells appears to be dominated by Th1 cells with high activation of macrophages (14). The cytokines from these two cell types cause TSS (2, 7).

In an apparently smaller percentage of humans, there appears to be a skewing toward dominance by Th2 cells. As much as 10% of the population of the United States may have atopic dermatitis caused by such Th2 skewing (2), but without the profound hypotension and shock of TSS. However, we recently described a small number of patients with what appears to be an anaphylactic or atopic type of TSS (32), the atopic type being without hypotension and shock. In the current study, we showed that the immortalized keratinocytes examined showed significant IL-33 production in response to both TSST-1 and SEB. IL-33 is an integral signaling molecule that promotes Th2 skewing (19). It is interesting that the source of the immortalized keratinocytes is not known to have had atopic dermatitis but is known to have had significant IgE-mediated allergies. Such an upregulation of IL-33 was not seen in the RNA-seq analysis performed on primary keratinocytes from a different person, indicating variability in the human population, such as is seen with atopic dermatitis susceptibility (2).

Finally, it is also known that group A streptococci are common causes of skin infections (2). We also know that all group A streptococcal strains which cause human diseases produce superantigens (2). Until now, we performed minimal studies with the two major streptococcal superantigens associated with serious diseases, namely, SPEA and SPEC (2, 4–6). In the current study, we showed that SPEA, a superantigen related to SEB and SEC (2, 7), and SPEC, a superantigen most closely related to TSST-1, simulate keratinocytes to produce the chemokine IL-8. The doses of SPEA and SPEC that were required for stimulation was approximately the same as the TSST-1 and SEB doses, suggesting that the pathways involved that led to activation were similar. This suggests that the expression levels of other chemokines are also likely to be upregulated by the streptococcal superantigens, just as they are for the staphylococcal superantigens.

In sum, our studies have shown that human keratinocytes, both primary and immortalized, are activated significantly by superantigens from both S. aureus and group A streptococci. The data suggest that the downstream effect of this activation is harmful inflammation, which may contribute to barrier dysfunction and disease.

## MATERIALS AND METHODS

### Superantigens.

All media and reagents used to prepare superantigens were maintained under pyrogen-free conditions. TSST-1, SEB, SPEA, and SPEC were purified by combinations of ethanol precipitations from culture fluids followed by thin-layer isoelectric focusing (33, 34). The superantigens that were thus purified were shown to be homogeneous by SDS-PAGE (33, 34). These same lots of superantigens have been used in studies of effects on HVECs (13).

### Primary human cells.

Human skin keratinocytes were isolated from discarded skin obtained from breast reduction surgery. Briefly, skin was disinfected with povidone-iodine (Betadine), washed with phosphate-buffered saline (PBS; 0.15 M NaCl, 0.005 M NaPO_4_, pH 7.2), and incubated overnight in dispase (20 μg/ml in Dulbecco’s modified Eagle’s medium [DMEM]) at 4°C. The epidermis was removed followed by trypsinization to dislodge the basal epithelial cells. After being spun and washed in PBS, the cells were plated in keratinocyte serum-free medium (KSFM) containing antibiotics (penicillin and amphotericin B [Fungizone]). Cells were expanded and frozen in aliquots at an early passage for experiments.

### Treatments of cells.

Primary keratinocytes were plated at 800,000 cells per dish in 10-cm-diameter tissue culture plates in KSFM. Media were changed for the cells daily. The plates were ∼80% confluent after 3 days, at which time they were treated in new media with 100 μg/ml TSST-1 or SEB. Cells were incubated at 37°C in a tissue culture incubator with 5% CO_2_ and were harvested for RNA after 6 h.

### RNA isolation and processing for RNA-seq.

Cells were homogenized in 1 ml of TRIzol reagent (Invitrogen). Total RNA from the aqueous phase was further purified using RNeasy columns (Qiagen). Transcription profiling using RNA-seq was performed by the University of Iowa Genomics Division using manufacturer-recommended protocols. Briefly, 500 ng of DNase I-treated total RNA was used to enrich for poly(A)-containing transcripts using beads coated with oligo(dT) primers. The enriched RNA pool was then fragmented, converted to cDNA, and ligated to sequencing adaptors containing indices using an Illumina TruSeq stranded mRNA sample preparation kit (catalog no. RS-122-2101; Illumina, Inc., San Diego, CA). The molar concentrations of the indexed libraries were measured using a model 2100 Agilent Bioanalyzer (Agilent Technologies, Santa Clara, CA), and the reaction mixtures were combined equally into pools for sequencing. The concentrations of the pools were measured using an Illumina library quantification kit (Kapa Biosystems, Wilmington, MA) and sequenced on an Illumina HiSeq 4000 genome sequencer using 150-bp paired-end SBS (sequencing by synthesis) chemistry.

### RNA-seq bioinformatic analysis.

At a minimum, three replicates were prepared per sample. Barcoded samples were pooled and sequenced, using an Illumina HiSeq 4000 genome sequencer in the Iowa Institute of Human Genetics (IIHG) Core Facility, to obtain a minimum of 30 million paired-end 150-bp reads per sample per ENCODE project standards. Reads were converted from the native Illumina BCL format to the fastq format and were processed with a standard RNA-seq pipeline, available through the open-source “bcbio-nextgen” project (https://github.com/chapmanb/bcbio-nextgen). This pipeline includes “best practices” approaches for quality control (QC), alignment, and read quantitation. The bcbio-nextgen pipeline runs qualimap, a computational tool that examines SAM/BAM alignment files and provides an overview of the data to enable detection of common QC problems. Reads were aligned against the hg38 reference genomes using the ultrarapid STAR aligner and, concurrently, pseudoalignment reads to the transcriptome using a Salmon aligner. Typically, 80% to 85% of RNA-seq reads were uniquely mapped to the reference. Samples that deviated in QC parameters were flagged and in some cases excluded from further analysis (such as principal-component analysis of gene expression), depending on the nature of the QC deviation. Following alignment, expression values were summarized at the gene and transcript levels. FeatureCounts was used for STAR-aligned data, while Salmon performed its own internal quantitation, yielding estimated counts and values in numbers of length-normalized transcripts per million (TPMs). Raw counts were used at the gene and transcript levels for analysis of expression of differentially expressed (DE) genes using *DESeq2* and a false-discovery-rate cutoff value of 0.05. The sets of DE genes were imported into iPathwayGuide commercial software to perform a sophisticated overrepresentation and perturbation analysis to generate reports of statistically enriched pathways, upstream regulatory genes, miRNAs, biological processes, diseases and functions, and interaction with metabolites and chemicals.

### Enzyme-linked immunosorbent assay (ELISA) of cytokines.

Immortalized human keratinocytes collected from a volunteer different from the donor from whom the primary cells were collected (35, 36) were cultured to confluence in triplicate wells of flat-bottom 96-well microtiter plates in KSFM in a 5% CO_2_ incubator at 37°C. At that time, the medium was changed to new KSFM containing doses of TSST-1, SEB, SPEA, or SPEC. Plates were incubated for an additional 6 h in the CO_2_ incubator. The plates were then placed in a −20°C freezer to stop the reactions. The next day, cytokine assays were performed. The assays were performed according to the specifications provided by the manufacturer (R&D Systems, Minneapolis, MN).

### Statistics.

ELISA data were analyzed by Student's *t* test analysis of unpaired data.

### Data availability.

Data determined in the work are available under GEO accession numbers GSM4711950 to GSM4711959 and GSE155775.
